# Intraoperative hemodynamic management during pancreatoduodenectomy – an analysis of 525 patients

**DOI:** 10.1007/s00423-025-03669-w

**Published:** 2025-04-08

**Authors:** Maximilian Dietrich, Tobias Hölle, Mattia Piredda, Manuel Feißt, Patrick Rehn, Maik von der Forst, Dania Fischer, Thilo Hackert, Jan Larmann, Christoph W. Michalski, Markus A. Weigand, Martin Loos, Felix C. F. Schmitt

**Affiliations:** 1 Medical Faculty Heidelberg, Department of Anesthesiology, Heidelberg University, Heidelberg, Germany; 2 Institute for Medical Biometry, Medical Faculty Heidelberg, Heidelberg University, Heidelberg, Germany; 3 Department of General, Visceral and Thoracic Surgery, University Hospital Hamburg-Eppendorf, Hamburg, Germany; 4 Department of General, Visceral and Transplantation Surgery, Medical Faculty Heidelberg, Heidelberg University, Heidelberg, Germany; 5 Department of Anesthesiology, Heidelberg University Hospital, Im Neuenheimer Feld 420, 69120 Heidelberg, Germany

**Keywords:** Pancreatic surgery, Hemodynamics, Fluid administration, Hemodynamic management

## Abstract

**Importance:**

Optimization of perioperative hemodynamic management during major pancreatic surgery can reduce postoperative complications.

**Objective:**

In this study, we aimed to investigate the effect of intraoperative hemodynamic management, in consideration of both anesthesiologic and surgery-related aspects on major short-term complications following partial pancreatoduodenectomy (PD).

**Design, setting and participants:**

Data of 525 patients undergoing PD between January 2017 and December 2018 at the Heidelberg University Hospital were retrospectively analyzed.

**Main outcomes and measures:**

Primary outcome was a composite of 90-day mortality, pancreatic fistula and completion pancreatectomy. Logistic regression was performed to estimate the impact of anesthesiologic and surgical factors. Furthermore, patients were stratified by the amount of fluid administered intraoperatively and the maximum catecholamine dose to examine the impact on the primary endpoint.

**Results:**

Using logistic regression analysis we demonstrated that epidural anesthesia was associated with a reduction in the occurrence of the combined endpoint (OR 0.568; CI 0.331–0.973), this effect was primarily driven by a lower rate of completion pancreatectomy. The intraoperative administration of fresh frozen plasma (FFP) doubled the odds of the occurrence of the primary endpoint (OR 2.238; CI 1.290–3.882). The comparison of patients with and without FFP transfusion showed that all components of the primary endpoint were more frequent in the FFP group. Complication rates in the stratified fluid groups showed a U-shaped curve with the least amount of complications in patients who received 6.5 to 8 ml/kg/h of intraoperative fluid. The comparison of maximum norepinephrine doses revealed the same pattern with the least complication rate in the low-intermediate dose range (0.05–0.08 µg/kg/min and 0.08–0.11 µg/kg/min).

**Conclusions and relevance:**

Epidural anesthesia had a beneficial effect on the rate of major surgical complications following PD, whereas intraoperative FFP transfusion showed a negative association. Intraoperative hemodynamic management appears to have a major impact on perioperative mortality and morbidity with a U-shaped relation for both fluid and vasopressor dose.

**Supplementary Information:**

The online version contains supplementary material available at 10.1007/s00423-025-03669-w.

## Background

Optimization of hemodynamic therapy during major surgery can reduce postoperative complications [1]. Especially during partial pancreatoduodenectomy (PD), an individualized hemodynamic management appears essential to minimize perioperative complications. On the one hand, excessive intraoperative fluid administration bears the risk of increased tissue edema formation. On the other hand, fluid loss must be adequately replaced to maintain adequate tissue perfusion [2]. Both tissue edema and hypoperfusion impair the healing of abdominal anastomoses and increases the risk of developing complications such as postoperative pancreatic fistula (POPF), consecutive abdominal sepsis and postoperative organ failure. Regarding the intraoperative dosage of vasopressors, a similar risk-benefit-ratio on tissue perfusion can be assumed, since postoperative organ dysfunction can be reduced by individualized blood pressure control [3].

There are several randomized controlled trials investigating different fluid management concepts with heterogeneous findings. Intraoperative normovolemic hemodilution led to an increased rate of pancreatic anastomotic complications, likely related to higher intraoperative fluid administration [4]. Weinberg et al. reported in a small randomized-controlled trial a reduced fluid balance and improved outcome using a cardiac output goal-directed therapy algorithm [5]. Intraoperative and postoperative fluid restriction in pancreatic surgery has generally not been shown to be beneficial. The studies conducted to date show contradictory results [6–10]. However, the comparability of these studies is limited by the use of different types of fluids, the heterogeneous interpretation of “restrictive” fluid therapy, and differences in the treatment of the control groups. In literature the relationship between postoperative complications and the amount of intraoperatively administered fluid is described as a U-shaped curve: Both restrictive and liberal fluid administration lead to a higher rate of complications following major abdominal surgery [11, 12].

Pancreatic surgery is predisposed to increased intraoperative blood loss due to the proximity of the pancreas to major vessels. Therefore, intraoperative hemodynamic therapy must be evaluated in conjunction with blood loss and transfusion practices. Different studies reported reduced long-term survival for patients who received perioperative blood transfusions [13]. However, blood transfusions are lifesaving in cases of severe hemorrhage and are still performed during a large proportion of major pancreatic surgical procedures [14]. Furthermore, most studies on transfusion-associated adverse events focus on red blood cell transfusion. There is only limited data on the effect of fresh frozen plasma (FFP).

In addition to surgical factors such as blood loss and the extent of surgical trauma, the anesthetic procedure also affects the cardiocirculatory system. Patients receiving epidural anesthesia had higher need for vasopressors without relevant impact on clinical outcomes [15]. Nevertheless, it needs to be considered evaluating intraoperative hemodynamic therapy.

In this retrospective analysis, we aimed to investigate the impact of intraoperative hemodynamic management with consideration of blood transfusions, as well as anesthesiologic and surgery-related aspects on major short-term complications following PD.

## Methods

### Study design and patient cohort

This study is a retrospective analysis of data from 525 consecutive patients who underwent partial pancreatoduodenectomy (PD) between January 1, 2017, and December 31, 2018 at the University Hospital Heidelberg. The study protocol was approved by the Heidelberg University Medical Faculty Ethics Committee (reference number: S-750/2019; Chairman Prof. Dr. Strowitzki) on 11.11.2019.

### Surgical procedure

Elective pylorus-preserving PDs, pylorus-resecting PDs, and classic Whipple procedures (cWhipple) were included. For anatomic reconstruction the following anastomoses were performed by default: double-layer end-to-side pancreaticojejunostomy, single-layer, end-to-side hepaticojejunostomy, and a double-layer, antecolic duodeno- or gastrojejunostomy.

### Anesthesiologic and hemodynamic management

Thoracic epidural anesthesia was established depending on the patient`s preference and in absence of contraindications. As a standard 10 ml of ropivacaine hydrochloride 0,5% (5 mg/mL) supplemented with 20 µg of sufentanil citrate was administered prior to surgery, followed by an intra- and postoperative continuous infusion rate of 6-10 ml/h Ropivacain 0,2% (2 mg/mL). Propofol 1%, Sufentanil and Rocuronium were used for the induction of anesthesia. After endotracheal intubation, an inhalational agent (Sevofluran or Desfluran) in combination with Sufentanil was used for anesthesia maintenance. The administration of fluids, blood products and catecholamines was determined by the care-giving anesthesiologist without a goal-directed therapy algorithm or any other restrictions.

### Data collection

The analyzed data were collected retrospectively from the clinical documentation system, especially from the anesthetist`s and surgeon`s documentation protocols. Demographics, comorbidities, anticoagulation, indication for the partial pancreatoduodenectomy, duration of surgery, extent of the surgical procedure, blood loss, anesthetic medication, the application of an epidural anesthesia, amount and type of given fluid (crystalloids, colloids, packed red blood cells (PBRC), FFP, albumin), urine output, fluid balance and maximum vasopressor dose were obtained.

Serum amylase, serum lipase, drain amylase, drain lipase, leucocyte count, platelet count, INR, C-reactive protein (CRP), albumin, creatinine, and lactate levels were collected from the laboratory documentation system. Laboratory values were evaluated from the day of surgery until postoperative day 14. The lowest intraoperative hemoglobin and the highest lactate were acquired from intraoperative blood gas analysis.

### Definition of postoperative pancreatic fistula (POPF)

POPF was defined according to the 2016 update of the International Study Group (ISGPS) classification [16]. Increase of drain amylase higher than > 3 times the upper limit of the institutional normal serum amylase activity without clinical consequences was defined as Biochemical Leak. If a change of the postoperative management was required (either drains left in place > 3 weeks or endoscopic or percutaneous procedures necessary) the fistula was graded type B. The necessity of reoperation or the occurrence of multiple organ failure and/or mortality attributable to the pancreatic fistula was classified type C.

### Study endpoints

The primary outcome was a composite of death from any cause until postoperative day 90, POPF (type B or C) and the necessity for completion pancreatectomy. Patients who underwent total pancreatectomy as part of the primary surgery were excluded from this analysis. Secondary outcomes comprised the single components of the primary composite endpoint, as well as ICU- and In-hospital stay.

### Statistical methods

Descriptive statistics and general visualization were performed on all relevant data. Metric data is reported as mean and standard deviation (SD), or as median and inter quartile range (IQR). Categorical values are reported as absolute and relative frequencies. A t-Test or Mann Whitney U-Test was performed to analyze metric data as appropriate. Categorical values were compared using a Chi-Squared-Test. The logistic regression was performed using a complete case analysis approach with only 26 patients excluded from the final analysis because of missing data. Variables included in the analysis were basic patient characteristics as well as major anesthesiologic management parameters. Forward selection was used to select variables for the final model. The Wald test was used to assess the significant influence of the parameter. Multicollinearity was examined with correlation analysis of all metric variables. The linear dependency of the outcome variable logit was assessed visually for each variable. The level of statistical significance was set at 0.05. Since this is a exploratory study, p-values has to be interpreted in a descriptive sense and are not adjusted for multiplicity. Statistical analysis was performed using the statistic software SAS version 9.4.

## Results

### Baseline characteristics and outcome

A total of 525 patients underwent elective pancreatic surgery in the studied period. Mean age of patients in the studied cohort was 62.7 years (SD 12.3) with a mean BMI of 24.8 kg/m² (SD 12.3). Baseline characteristics are shown in Table 1.

**Table 1:**
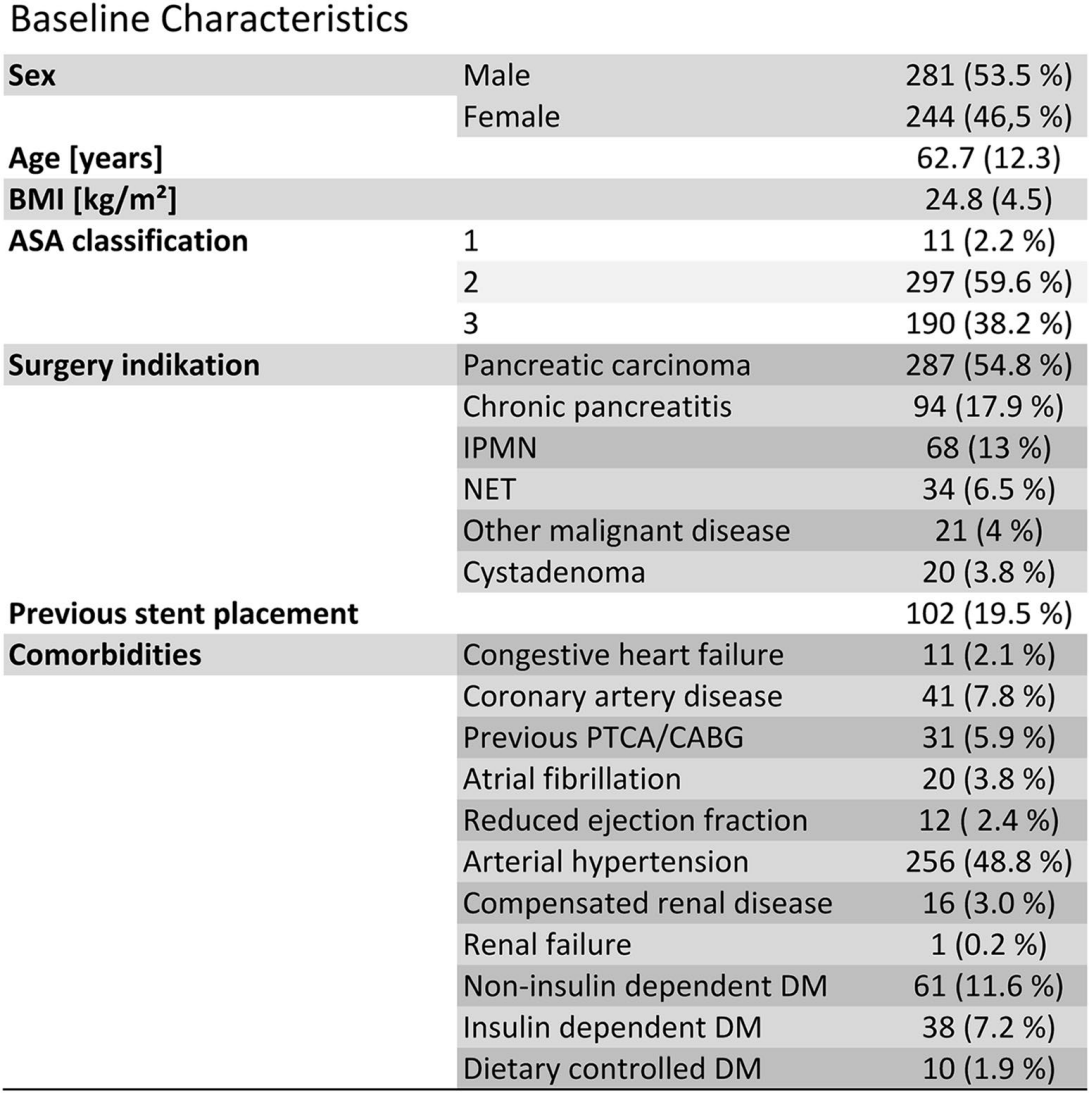
Baseline characteristics of the collective: reported as mean and standard deviation or absolute and relative frequencies. ASA classification: American society of anaesthesiologists physical status classification IPMN: intraductal papillary mucinous neoplasms, kg: kilogram; m²: square meter, NET: neuroendocrine tumor, DM: diabetes mellitus, PTCA: Percutaneous transluminal coronary angioplasty, CABG: Coronary artery bypass graft surgery

A comparison of total intravenous anesthesia (TIVA) vs. inhaled anesthetic was not included because of the low number (*n* = 2) of patients receiving a TIVA in the studied period. The mean duration of surgery was 5.6 h (SD 1.6) with an associated 7.2 h of mean duration of anesthesia. Relative amount of fluids administered were calculated using the duration of anesthesia.

A total of 74 (14.2%) patients met the composite endpoint, divided into 15 (2.9%) deaths, 24 (4.6%) completion pancreatectomies, and 60 (11.4%) clinically relevant pancreatic fistulas. The specific reasons why the patients needed completion pancreatectomy after PD were acute necrotizing pancreatitis in 7 patients (29.2%), pancreatitis in 3 patients (12.5%), uncontrollable POPF in 3 patients (12.5%), hemorrhage in 3 patients (12.5%) or others in 8 patients (33.3%). All surgical and outcome parameters are shown in Table 2.

**Table 2:**
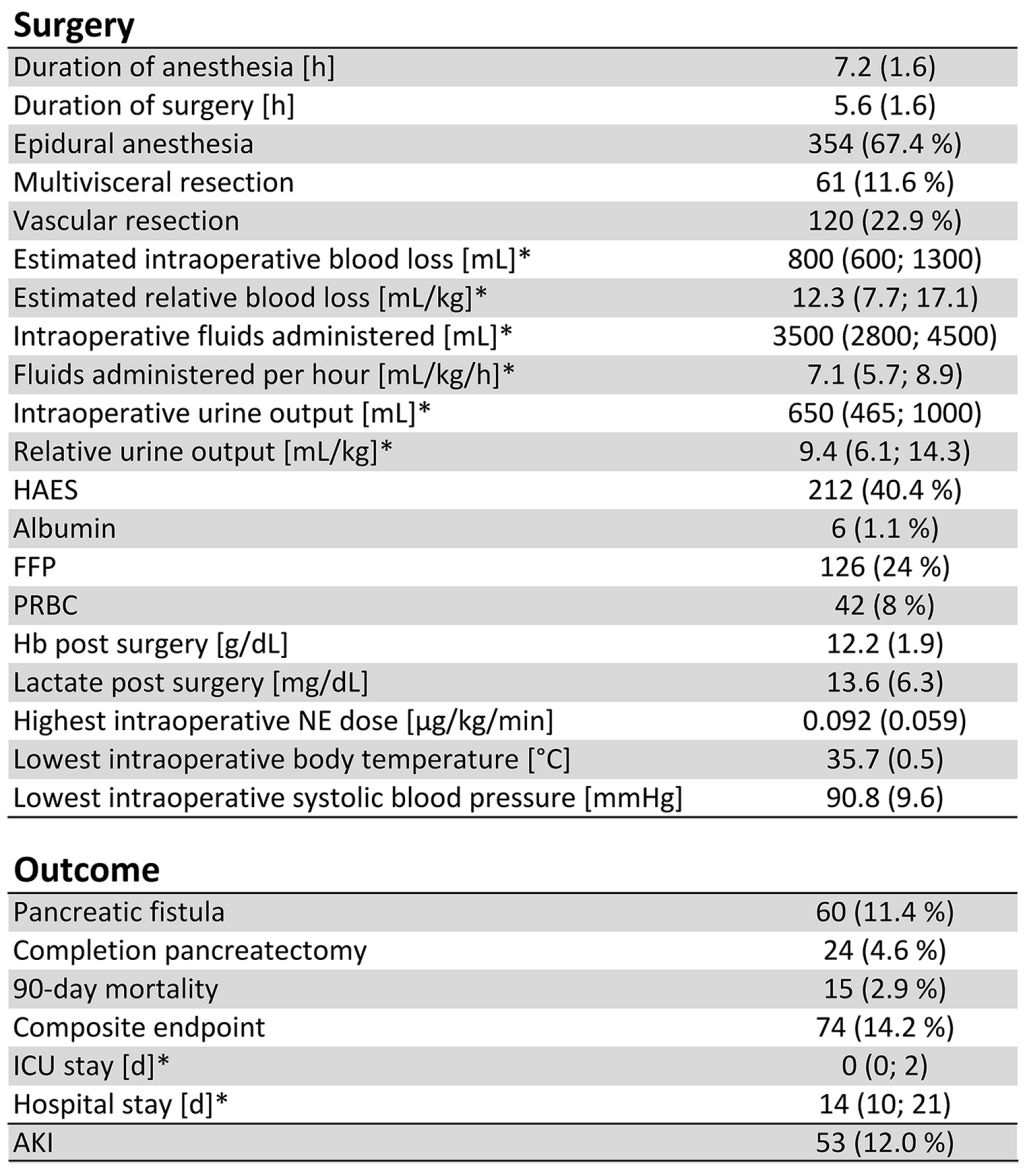
Surgical parameters and endpoints: metric values are reported as mean and standard deviation or as median and interquartile range (indicated by *). Categorical values are reported as absolute and relative frequencies. HAES: Hydroxyethyl-starch; FFP: fresh frozen plasma; PRBC: packed red blood cells; hb: haemoglobin; NE: norepinephrine; ICU: intensive care unit; AKI: acute kidney injury; h: hours; mL: millilitre; mL/kg: millilitre per kilogram; mL /kg /h: millilitre per kilogram per hour; g /dL: gram per decilitre; Mg /dL: Mper decilitre; Μg /kg /min: microgram per kilogram per minute; °C: degrees celsius; MmHg: millimetres of mercury; D: days

## Logistic regression

The final logistic regression revealed a significant influence of BMI, the presence of an epidural anesthesia, and the administration of FFP on the occurrence of the composite endpoint (Table 3).

**Table 3:**
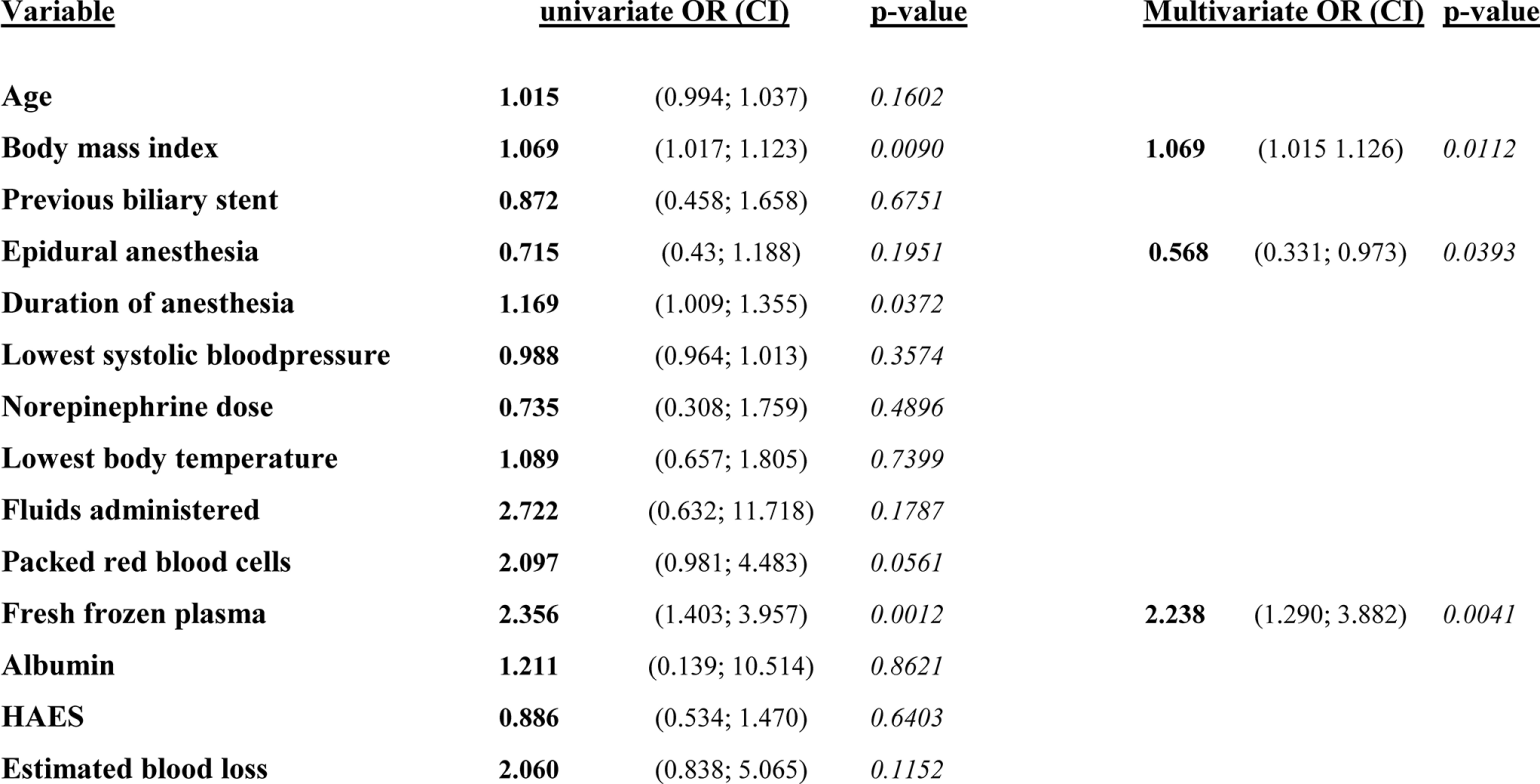
Logistic regression results for the composite endpoint: endpoint was set as a combination of 90-day mortality, relevant pancreatic fistula or the need for completion pancreatectomy. Level of significance was set at 0.05. Unadjusted univariate analysis is shown as well as final multivariate model. Forward selection was used for variable selection for final model. Odds ratios (OR) with corresponding 95% confidence interval (DI) are reported. HAES: Hydroxyethyl-starch

The use of epidural anesthesia had a beneficial effect on the development of complications (OR 0.568; CI 0.331–0.973). The administration of FFP was associated with doubled odds for the occurrence of the primary composite endpoint (OR 2.238; CI 1.290–3.882) and an increase in BMI increased the odds by 1.069 per BMI point (CI 1.015–1.126). Other factors like the overall amount of fluids or the duration of anesthesia did not reveal a significant harm increase or mitigation.

### Epidural anesthesia was associated with reduced incidence of completion pancreatectomy

**Fig. 1:**
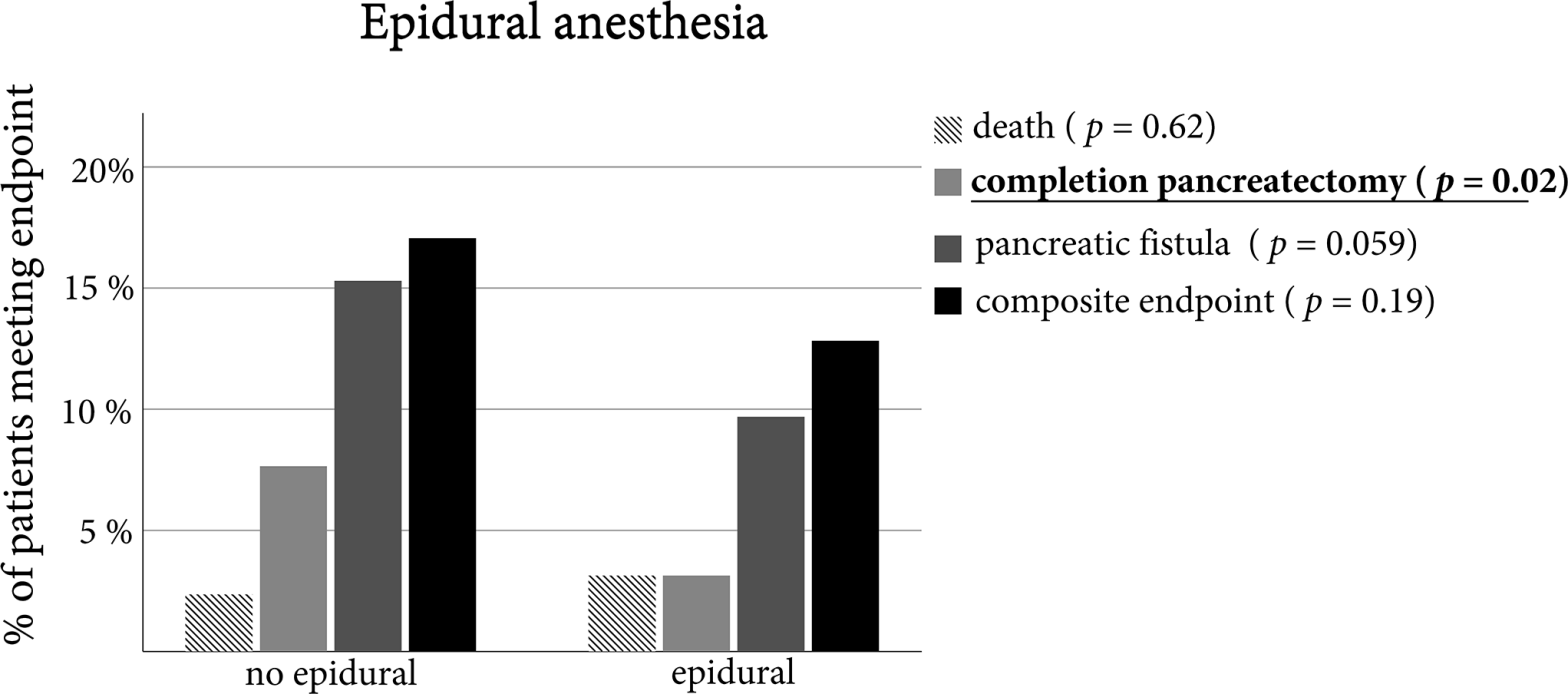
Relative frequencies of major complications for patients with and without epidural anaesthesia. Statistical comparison was performed using a Chi-Squared Test. Results were considered significant at a *p* < 0.05 and were written in bold

The unadjusted distribution of the endpoint and the respective individual factors were examined, due to the categorical scale of the endpoint. The purpose of this investigation was to determine whether the effect was primarily due to one aspect of the endpoint or to the summation of the influences (Fig. 1). We found that the reduction of the endpoint was primarily triggered by a reduction in the need for completion pancreatectomies, the lower occurrence of fistulas did not reach significance. Epidural anesthesia increased the demand for vasopressors by 0.037 µg /kg /min (no epidural 0.67 µg /kg /min (SD 0.052) vs. epidural 0.104 µg /kg /min (SD 0.058)). On the other hand, epidural anesthesia did neither affect the occurrence of acute kidney injury (no epidural 17/148 (11.5%) vs. epidural 36/295 (12.2%)) nor the duration of hospital or ICU stay in our collective.

### Patients who received FFP had a higher of postoperative pancreatic fistula, completion pancreatectomy and death

**Fig. 2:**
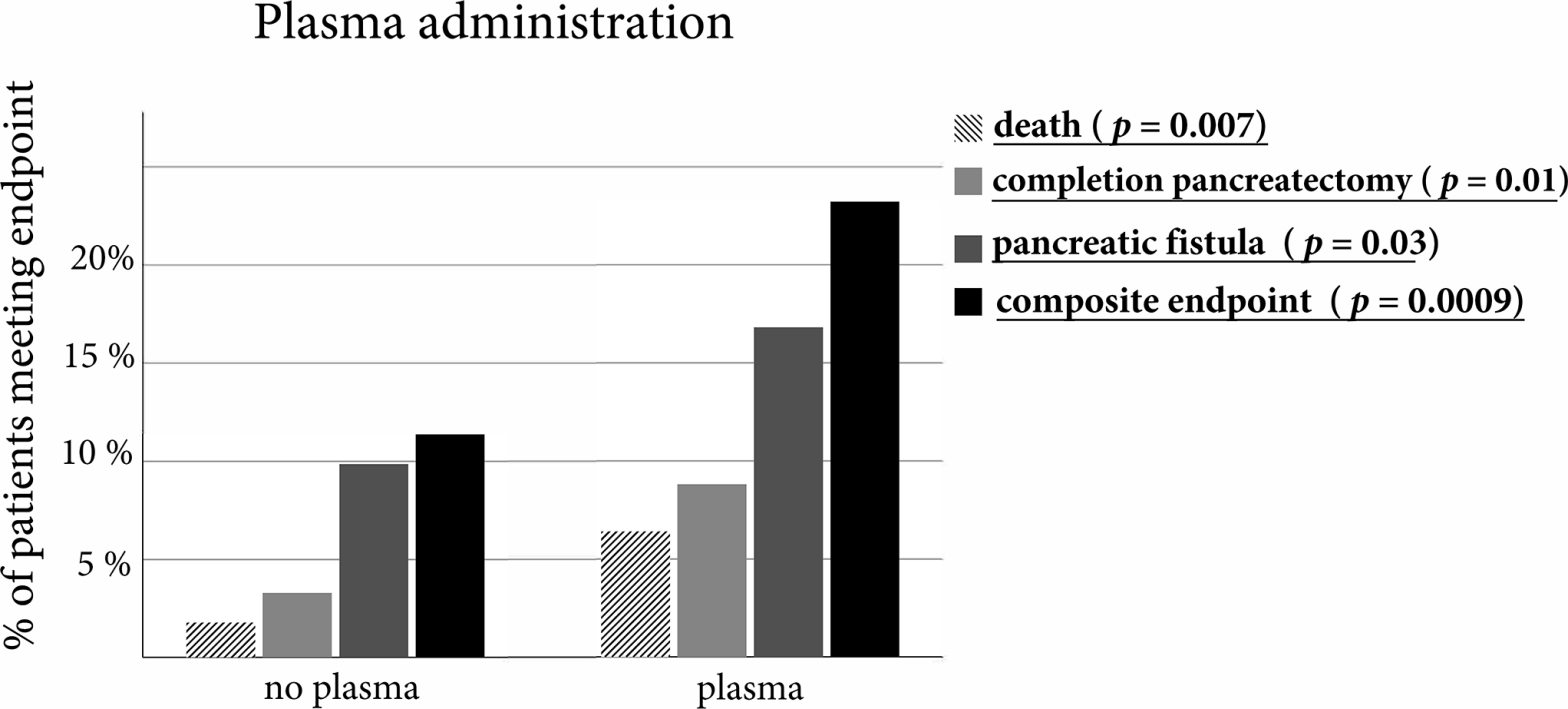
Relative frequencies of major complications for patients with and without fresh frozen plasma transfusion. Statistical comparison was performed using a Chi-Squared Test. Results were considered significant at a *p* < 0.05 and were written in bold

**Fig. 3:**
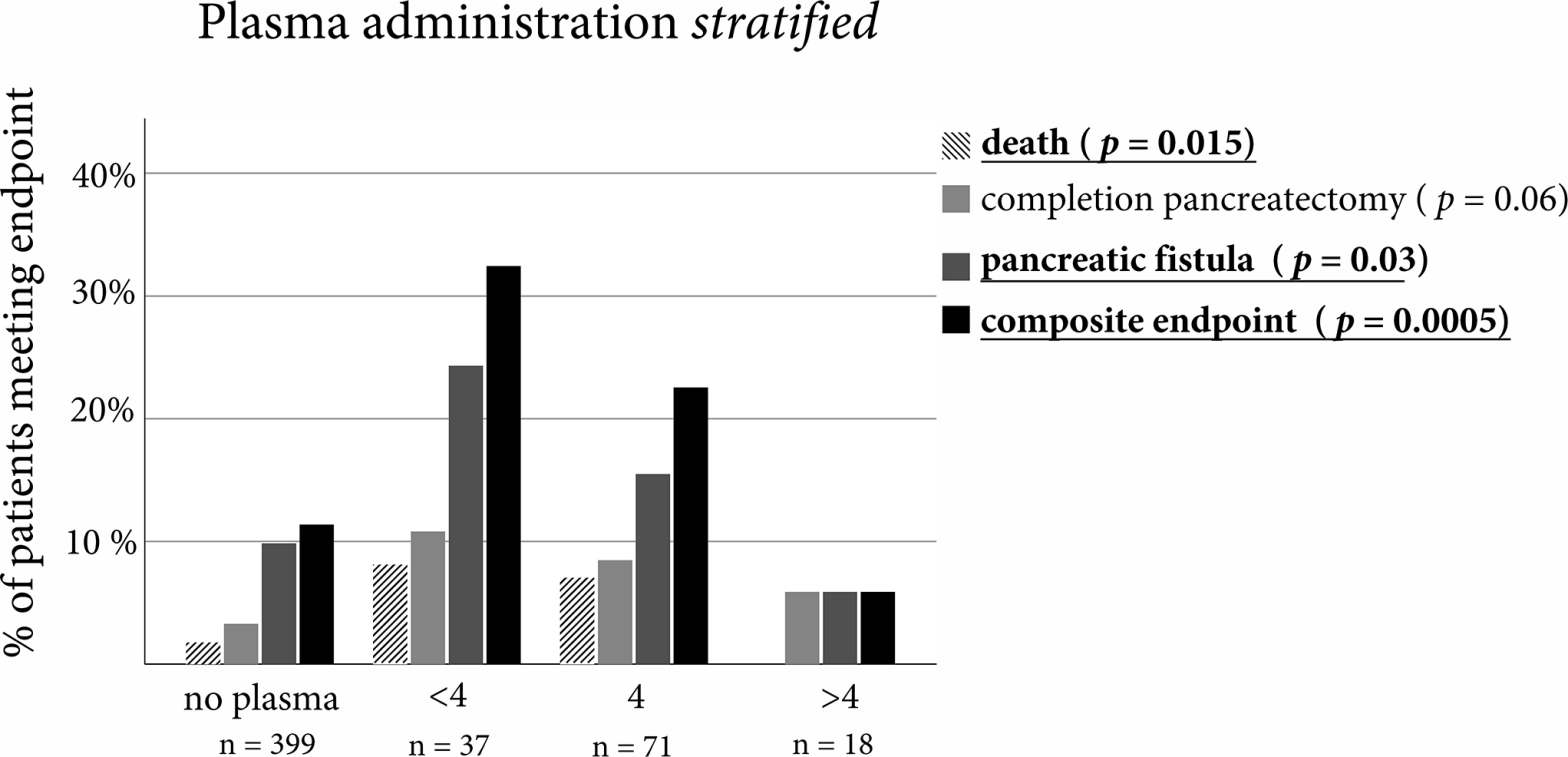
Relative frequencies of major complications for patients who received fresh frozen plasma (FFP) transfusion by amount. Patients were stratified into patients receiving no transfusion (0), less than 4 (< 4), exactly 4 units (4) and more than 4 units of FFP. Statistical comparison was performed using a Chi-Squared Test. Results were considered significant at a *p* < 0.05 and were written in bold

An analysis of the patients who received plasma showed that the plasma group had significantly more overall complications, including death (Fig. 2). Patients receiving FPP hat significantly higher intraoperative blood loss (*p* < 0.001; no FPP: 750 mL (IQR: 500; 1000); FFP 1500 mL (IQR: 1000; 2200)). A further stratification into high (> 4), intermediate (4), and low (< 4) amount of plasma administered showed that the effect seems to be predominantly present in the low plasma group (Fig. 3). The effect of the transfusion of PRBC on the primary endpoint did not reach significance in the univariate logistic regression analysis.

### The relation of both intraoperative fluid and vasopressor dose to postoperative complications follows a u-shaped curve

**Fig. 4:**
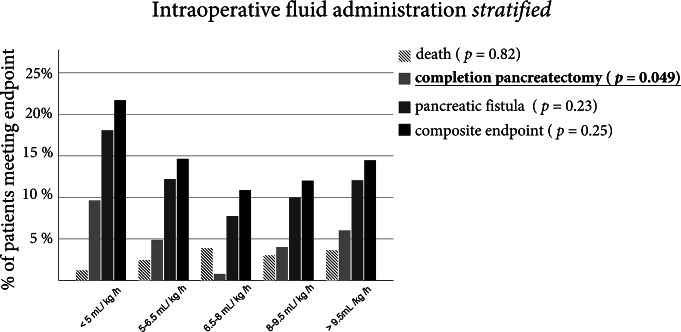
Relative frequencies of major complications for patients stratified by amount of fluids administered. Patients were grouped into five categories consisting of roughly equal sized subpopulations. Statistical comparison was performed using a Chi-Squared Test. Results were considered significant at a *p* < 0.05 and were written in bold

We further stratified the amount of fluids that the patient received into 5 groups containing roughly the same number of patients (for further information see supplementary Table 1). The complications in the different groups showed a U-shaped curve with the least amount of complications in the 6.5 to 8 ml/kg/h group. However, this difference in distribution was only significant for the completion pancreatectomy (Fig. 4).

**Fig. 5:**
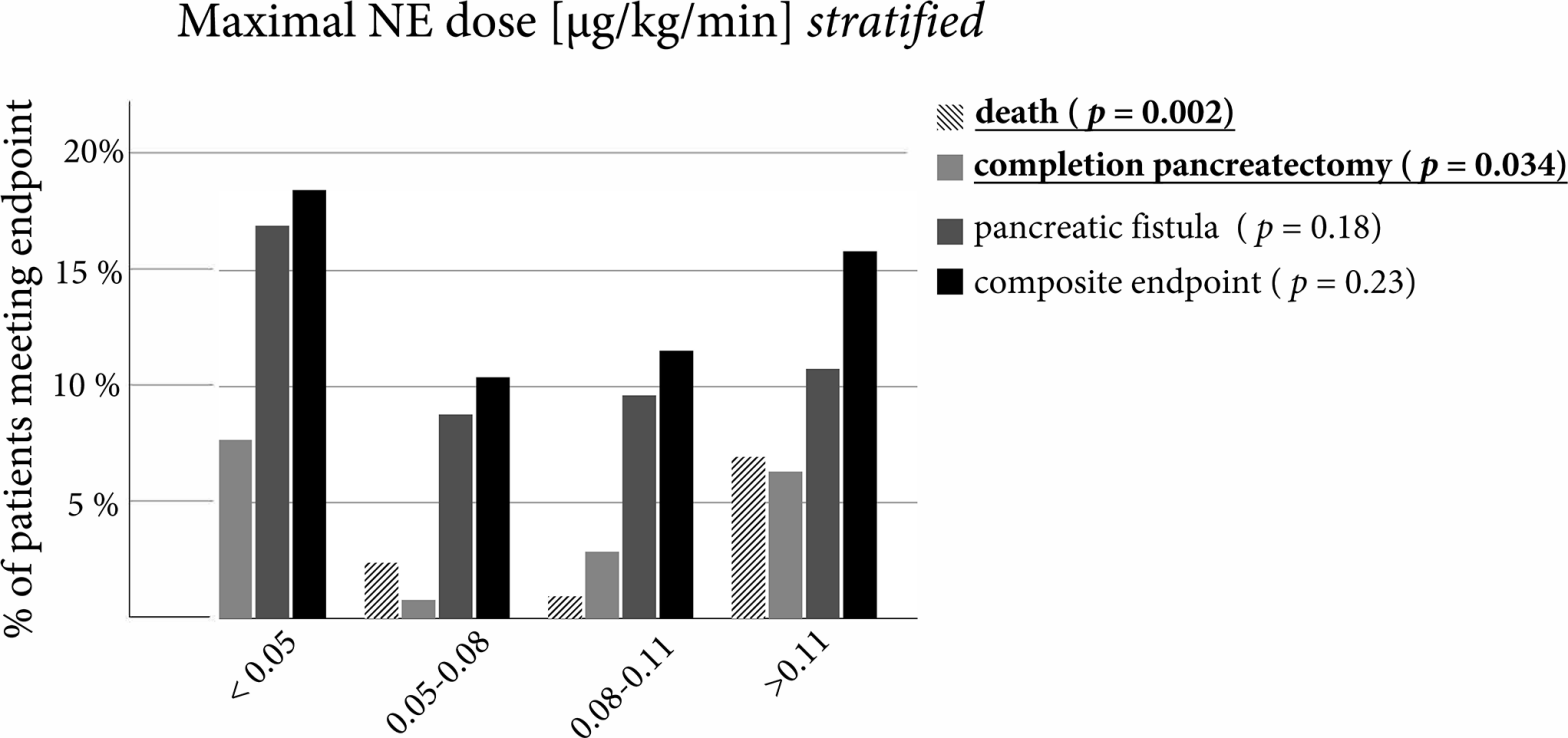
Relative frequencies of major complications for patients stratified by maximum norepinephrine dose. Patients were grouped into four categories consisting of roughly equal sized subpopulations. Statistical comparison was performed using a Chi-Squared Test. Results were considered significant at a *p* < 0.05 and were written in bold

For the further analysis of catecholamine influence, we stratified the cohort into 4 roughly equal sized sub cohorts. While the composite endpoint, pancreatic fistula and mortality did not show a significantly different distribution, the differences in the rate of completion pancreatectomy were significant with the highest incidence of completion pancreatectomy in the low dose group (Fig. 5).

## Discussion

In this retrospective analysis, we investigated the influence of hemodynamic and perioperative anesthesiologic management on major complications and mortality in a large cohort of patients undergoing PD. As primary endpoint, we chose a composite outcome of death, clinically relevant POPF, and the necessity for completion pancreatectomy. Logistic regression showed that a higher BMI and the intraoperative administration of FFP were associated with an increased incidence of the primary composite endpoint. Patients who did not receive FFP had a significantly lower rate of each component of the primary composite outcome. The opposite was observed for the use of epidural anesthesia, which reduced the occurrence of the above-mentioned complications. Patients who underwent epidural anesthesia during PD had a significantly lower rate of completion pancreatectomy. In line with previous publications about fluid management in major abdominal surgery, we found a U-shaped relation curve between the intraoperative fluid dose and perioperative major complications [12]. This pattern was also observed for the maximum intraoperative catecholamine dose.

The in-hospital mortality and morbidity following pancreatic surgery is inversely associated with hospital volume. In high-volume centers in Germany a mortality rate of ~ 6% has been described [17]. In comparison, the observed mortality rate in the present analysis was very low. The impact of a higher BMI on the incidence of major complications following pancreatic surgery is in line with the results of previous studies. High BMI is a well-known risk factor for adverse outcomes following PD [18, 19]. For the use of perioperative blood transfusions different adverse effects are described in literature. In critical care and elective general surgery, plasma transfusions were associated with postoperative infectious complications [20–22]. In addition, long term survival of patients who received blood transfusions during pancreatic cancer surgery has been reported to worsen [13]. In this analysis, there was a significant detrimental effect of intraoperative FFP administration, which was associated with doubled odds for the occurrence of the primary composite endpoint. Surprisingly, this effect was more pronounced in patients receiving ≤ 4 FFPs and not observable in patients receiving ≥ 4 units as it is the case in massive transfusion situations. For PRBCs we did not prove a significant effect on postoperative complications, however a trend was observable in the univariate regression analysis. Therefore, a detrimental effect of intraoperative transfusions in general cannot be ruled out. However, a bias due to confounding by indication cannot be excluded, since patients with greater surgical trauma and more comorbidities are naturally more likely to require intraoperative transfusion. In this study median blood loss was double the amount in patients receiving FPP compared to patients who did not receive FFP. It remains unclear whether FFP transfusion or the situation leading up to the transfusion caused the effect. As these are retrospective data, the indication for FFP administration cannot be reconstructed. The indication for the administration of FFP was set individually by the treating physician and therefore cannot be determined retrospectively. The current recommendation is no administration of FFP for volume substitution; to the authors’ knowledge, a prospective randomized study of FFP administration compared with crystalloid solution does not exist. Nevertheless, our data support a restrictive transfusion strategy.

Epidural anesthesia was used in most patients for perioperative analgesia. Various beneficial effects of an epidural anesthesia besides pain control have been shown, to name the most important: reduction of sympathetic activity and thereby improved perioperative organ function, decreased cardiac morbidity and mortality, as well as amelioration of gut injury [23]. However, hemodynamic effects of epidural anesthesia need to be considered [24]. In the present analysis epidural anesthesia was significantly associated with a reduction in the incidence of the primary outcome. This effect can be explained primarily due to the lower rate of completion pancreatectomies in patients, in whom epidural anesthesia was applied.

For both the intraoperative amount of administered fluid and the maximal vasopressor dose a U-shaped relation to the incidence of the primary composite outcome was observed. Our findings correspond to these of Adrianello et al., who observed an increased POPF rate in patients with a near zero fluid balance, as well as in patients undergoing liberal fluid administration [11]. Miller et al. also reported a U-shaped pattern for the correlation of intraoperatively given fluid and postoperative complications in a large cohort of patients undergoing non-cardiac surgery. However, the sample of Miller et al. included a variety of different surgical procedures, the majority of which are not transferable to complex pancreatic surgery [12]. Our data suggest the lowest complication rates with an amount of given fluid per hour and a maximal intraoperative vasopressor dose of 6.5-8 ml/kg/h and 0,05 − 0,08 µg/kg/min, respectively. However, these values should not be considered as the optimum for all patients in general, but the results underline that hemodynamic management is more complex than the frequently asked question liberal versus restrictive. It should also be considered that epidural anesthesia increases the need for catecholamines to mitigate the vasodilatory effects. Based on physiological principles hemodynamic therapy needs to be titrated individually for each patient with the aim to optimize tissue perfusion.

Several limitations need to be considered. First, this was a retrospective analysis of patients who underwent PD. Data was therefore collected from our clinical documentation system and the perioperative documentation protocols. Due to the form of documentation a time-weighted analysis of the vasopressor dose during the operation was not possible. However, the applied maximal dose is reproducible for retrospective analyses. Another limitation of our study is the lack of data on key preoperative risk factors for POPF, such as pancreatic duct diameter and gland consistency, which were not routinely assessed as part of our standard preoperative evaluation. These factors are known to influence POPF development and could also interact with the effects of hemodynamic management. Further it was a monocenter analysis of patients undergoing PD in a high-volume center with more than 250 PD procedures per year. It is therefore questionable whether the findings can be applied to smaller centers.

## Conclusion

Intraoperative hemodynamic management has a major impact on mortality and morbidity following PD. Both fluid and vasopressor dose follow a U-shaped relation to the incidence of the composite primary endpoint of POPF, completion pancreatectomy and death. The intraoperative transfusion of FFP was associated with an increased risk of postoperative major complications, while epidural anesthesia revealed a beneficial influence.

## Electronic supplementary material

Below is the link to the electronic supplementary material.

Supplementary Material 1

Supplementary Material 2

## Data Availability

No datasets were generated or analysed during the current study.

## Declarations

### Assistance with the article

None declared.

### Presentation

None declared.
