# Supplementary figures and images for: Intraoperative hemodynamic management during pancreatoduodenectomy – an analysis of 525 patients
Source: Langenbecks Arch Surg. 2025 Apr 8;410(1):123. doi: 10.1007/s00423-025-03669-w (PMC11978697; doi:10.1007/s00423-025-03669-w)

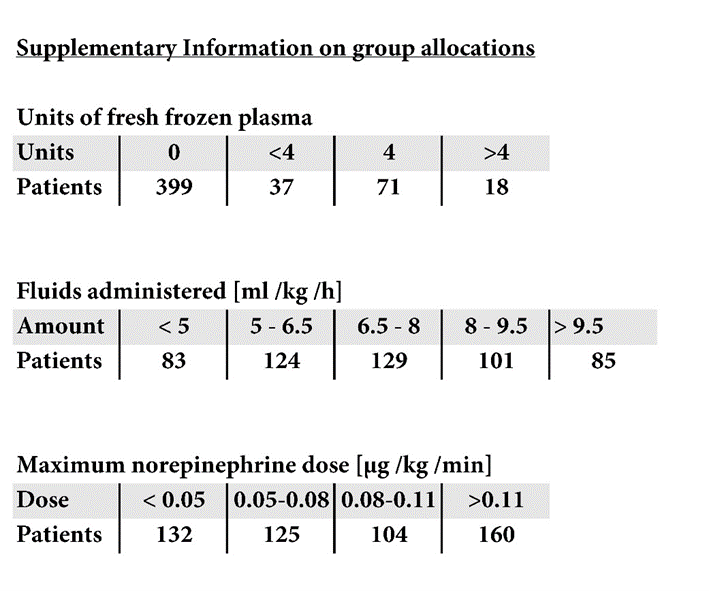

Supplement: Supplementary file 1 — Supplementary Material 1 [file 423_2025_3669_MOESM1_ESM.png]
